# Gene knockdown by RNAi in the pea aphid *Acyrthosiphon pisum*

**DOI:** 10.1186/1472-6750-7-63

**Published:** 2007-09-28

**Authors:** Stéphanie Jaubert-Possamai, Gaël Le Trionnaire, Joël Bonhomme, Georges K Christophides, Claude Rispe, Denis Tagu

**Affiliations:** 1 INRA, Agrocampus Rennes, UMR 1099 BiO3P (Biology of Organisms and Populations applied to Plant Protection), F-35653 LE RHEU, France; 2 Department of Biological Sciences, SAF Building, Imperial College London, UK

## Abstract

**Background:**

RNA interference (RNAi) is a powerful method to inhibit gene expression in a sequence specific manner.

**Results:**

Here, we described the development of RNAi by micro-injection of double-stranded RNA (dsRNA) in the pea aphid *Acyrthosiphon pisum*. Injection of dsRNA into whole aphid body induced the silencing of two marker genes with different expression patterns: the ubiquitously expressed *Ap-crt *genes encoding a calreticulin and the gut specific *Ap-cath-L *gene encoding a cathepsin-L. Time-course analysis of the silencing showed similar temporal patterns for both genes: inhibition started at 1 day after injection, reached its maximum at 5 days and stopped at 7 days. A comparable 40% decrease of gene expression was observed for *Ap-crt *and *Ap-cath-L*.

**Conclusion:**

The pea aphid is the first Hemipteran insect for which genome sequence will be available soon. The gene knockdown technique developed in this study will be an essential post-genomic tool for further investigations in aphidology.

## Background

Double-stranded RNA interference (dsRNAi) is a powerful strategy to transiently inactivate the expression of targeted genes. Injection of exogenous dsRNA triggers sequence-specific degradation of the target endogenous mRNA in the target cells/organisms. Dicer RNaseIII-type enzymes cleave cytoplasmic dsRNAs into small interfering RNAs (siRNAs) duplexes composed of approximately 21 nucleotides (nt). siRNAs duplexes are incorporated into a multiprotein RNA-inducing silencing complex (RISC) where the antisense strand guide RISC to its homologous target mRNA for endonucleolytic cleavage. This mechanism leads to the degradation of the targeted mRNA [1]. dsRNA mediated RNAi has emerged as one of the most promising tool to study gene function particularly in organisms for which stable transgenesis is not available. Exogenous introduction of dsRNA has been developed mainly in invertebrates such as nematodes [2,3] and insects [4,5]. dsRNA can be delivered to insects through artificial feeding [6] or microinjection [5]. Artificial feeding is a non-disrupting technique preserving the integrity of the treated animals. However, the precise amount of up-taken dsRNA cannot be monitored. Microinjection is widely used in multiple insect species such as mosquitoes, beetles, honey bees and grasshoppers [4,5,7-9]. In these insects, RNAi knockdown has been developed for various genes encoding for developmental proteins [10], salivary gland proteins [11], proteins involved in host-insect interaction [12], hormone receptors [13,14], or gut enzymes [6,15].

Aphids are considered as one of the main animal pests for agriculture. Damages caused by aphids are mainly due to their tremendous demographic potential and their adaptation to the modifications of environmental conditions. The International Aphid Genomic Consortium has selected the pea aphid *Acyrthosiphon pisum *as the model aphid species for developing genomic resources such as ESTs or genome sequencing [16,17]. In the perspective of the future accessibility of the pea aphid genome sequence, functional analyses tools such as inactivation of gene expression will be essential for further investigations. Stable transformation of insects is still limited to few model species (e.g. *Drosohila*, *Anopheles gambiae, Bombyx mori*...). Furthermore, this technique would probably be very difficult to develop in aphids because of the low amount of sexual eggs produced and the requirement of diapause after egg production. Transitory gene knockdown through dsRNAi offers an opportunity to address the role of aphid genes. Injection of siRNA has already been reported in the pea aphid to knockdown a gene expressed in salivary glands [18]. We described here an alternative RNAi strategy by injecting long dsRNA instead of siRNAs and by triggering different tissues. The development of RNAi technique in a new species (i.e. the pea aphid) requires the selection of appropriate marker genes. Few criteria define marker genes, which should show a significant RNA expression level (to facilitate the detection of RNA accumulation), not give a lethal phenotype when inactivated (to characterise the inhibition of gene expression) and show different spatial expression profiles to test the sensitivity of various tissues to RNAi. Based on these criteria, two *A. pisum *marker genes have been selected with different tissue expression: one ubiquitously expressed encoding a calcium binding protein (calreticulin) and another specifically expressed in the gut encoding a cysteine protease, a cathepsin L [19]. For each gene, dsRNAs of approximately 300–400 nt have been synthesised. Their injection in the aphid body led to the inhibition of approximately 40% of targeted genes expression for five days following the injection.

## Results

### Cloning and expression profiles of *A. pisum *calreticulin and cathepsin L genes

**Figure 1:**
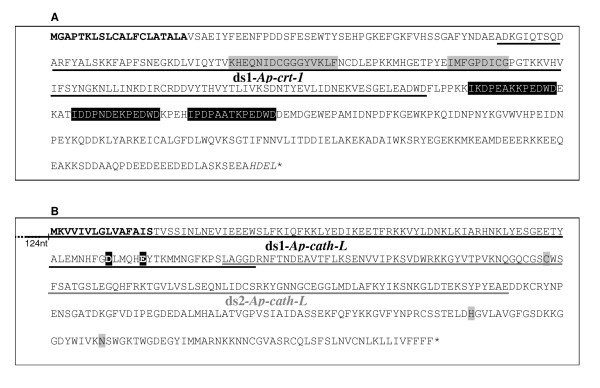
**Deduced amino-acid sequences of calreticulin (Ap-CRT-1) and cathepsin-L (Ap-cath-L) from *Acyrthosiphon pisum***. A. Deduced protein sequence of AP-CRT-1. Secretion signal peptide of 20 AA predicted by PSORTII [20] is bold, endoplasmic reticulum retention peptide "HDEL" is italicised, signature motifs of the calreticulin family are in black boxes, conserved repeated calreticulin sequences are in grey boxes. The location of the gene sequence used to synthesize dsRNA ds1-*Ap-crt-1 *is underlined. B. Deduced sequence of AP-CATH-L. Secretion signal peptide of 15AA is bold, conserved residues of active site are in black boxes and conserved carboxylic side chains important for acid autocatalytic processing of L cathepsins are in grey boxes. The location of the gene sequence used to synthesize dsRNA ds1-*Ap-cath-L *and ds2-*Ap-cath-L *are underlined in black and grey, respectively.

The *A. pisum *ESTs database [16] was screened in order to identify full coding sequences (CDS) of calreticulin and cathepsin-L. The CTG_AP_197.5-ApAL3SD-I-B1 contig was identified as encoding for a 408 amino acids (AA) calreticulin (Ap-CRT, Fig. 1A) and the CTG_AP_320.2-ID0AAA18AE10RM1 contig for a 341 AA cathepsin-L (Ap-cath-L, Fig. 1B). The sequences resulting from *in silico *assembly of ESTs were confirmed by sequencing of the corresponding cDNAs (data not shown).

The number of *Ap-crt *and *Ap-cath-L *gene copies in the pea aphid genome was investigated by assembling matching genomic sequences retrieved from the trace archives of the pea aphid genome. Two different calreticulin genes (*Ap-crt-1 *and *Ap-crt-2*) and one single *Ap-cath-L *gene were found. These *in silico *results were confirmed by Southern-blot analyses (data not shown). No EST was identified for *Ap-crt-2*, suggesting a low expression level for this gene.

No trace was identified for the 3' end of *Ap-crt-2 *and the incomplete CDS we obtained for this gene covered approximately 86% of the full CDS. Analysis of the corresponding partial CDS in *Ap-crt-1 *and *Ap-crt-2 *showed that these two genes shared 89% of nucleotide identity suggesting that both genes would be targeted by the same dsRNA. The expression of both calreticulin genes has thus to be analysed simultaneously to reflect the global efficiency of RNAi knockdown. Therefore, all the PCR primers we used for following analysis of calreticulin genes expression were designed in order to cross hybridised between *Ap-crt-1 *and *Ap-crt-2*. In the following experiments, both calreticulin genes are named as *Ap-crt*.

The temporal expression of *Ap-crt *and *Ap-cath-L *was investigated by RT-PCR on RNA extracted from the four instar stages and two adult morphs (parthenogenetic females and sexual females). Both genes were shown to be transcribed at all developmental stages (data not shown) indicating that any morph of the life cycle can be targeted to initiate RNAi. However, because of size restriction, injection was not possible for the first and second instars (L1, L2 respectively).

### Injection of dsRNA

Injections of dsRNA were performed on the third instar (L3) stage. The developmental stage of injected aphids was synchronised by collecting newly born first instar (L1) over a period of 24 h. 96 h later, after they all reached the third instar (L3), these larvae were immobilised on ice and dsRNAs were delivered into haemolymph by microinjection through a glass needle at slow speed. The injection was realised dorsally in the middle of the abdomens. Melanisation of the cuticle of the living aphids was observed at the injection point. A mortality of ranging from 30 to 45% was obtained in the two days following injection. Similar mortality was observed between individuals injected with a same volume of water, the control bacterial dsRNA *LacZ *or dsRNAs of interest (Table 1) indicating that aphid death was not related to gene silencing. Injecting less than 46 nl per aphids clearly reduced the mortality to approximately 13% indicating that the mortality we observed was partly due to the disruptive effect of injection itself but also by the high injection volume used to deliver dsRNA. However, in order to optimise the RNAi efficiency, we maximised the amount of delivered dsRNA by using 46 nl as the injection volume.

**Table 1:** Mortality induced by different volumes of injection. The disruptive effect of injection volume was estimated by testing three different volumes (46, 23 and 5 nl) of cathepsin-L dsRNA, bacterial LacZ control dsRNA, or water. For each tested volume, 20 L3 were injected and the mortality within the two days following the injection was measured and expressed as a percentage of injected aphids.

**Injected volume**	**Injected material**	**Mortality (%)**
**46 nl**	*ds1-Ap-cath-L*	45
	*ds-LacZ*	38
	H_2_O	29
**23 nl**	*ds-LacZ*	11
	H_2_O	13
**5 nl**	*ds-LacZ*	16
	H_2_O	14

### Silencing of calreticulin

**Figure 2:**
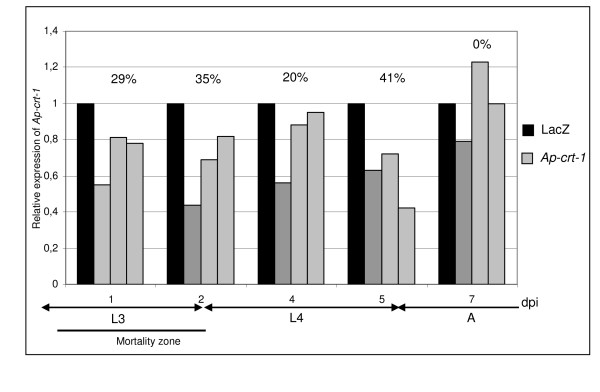
**Knockdown of *Ap-crt-1 *expression**. *Ap-crt-1 *mRNA level in control *LacZ *and ds*Ap-crt-1 *injected aphids were monitored by qRT-PCR over seven days after injection (dpi). Each kinetic point was performed in triplicate on 7–10 pooled aphids. For each sample, *Ap-crt-1 *transcripts level was normalised against *Rpl-7*. Normalised *Ap-crt-1 *expression in ds1*Ap-crt-1 *aphids was expressed as the proportion of the expression recorded in the *Lac-Z *control. Average percentages of *Ap-crt-1 *mRNA depletion are indicated. Developmental status of aphids is indicated below x axes.

A 434 nt dsRNA (ds1*Ap-crt*) corresponding to the 5' part of *Ap-crt-1 *CDS was designed for *Ap-crt *knockdown (Fig. 1). Analysis of the homologous sequence in *Ap-crt-2 *revealed that *Ap-crt-1 *and *Ap-crt-2 *were 87% identical for this region suggesting that both genes would be knockdowned by ds1*Ap-crt-1*. The efficiency of the inhibition of *Ap-crt *expression in injected aphids was investigated by quantitative RT-PCR (qRT-PCR) over seven days. For each time point, qRT-PCRs were performed on triplicates (3 pools of 7 to 10 injected aphids). As a negative control, aphids were injected with *LacZ *dsRNAs and qRT-PCR data were expressed relatively to the *RpL7 *internal control gene encoding for a ribosomal protein [21]. Results of the *Ap-crt *expression in ds*Ap-crt-1 *and control injected aphids are presented in Figure 2. Despite a high variability between the replicates, an unambiguous decrease of *Ap-crt *transcripts was observed in aphids injected with ds*Ap-crt-1*. *Ap-crt *expression was reduced by 29 ± 14% one day after injection (dpi). The maximum reduction of 41 ± 15% of the transcript level occurred at 5 dpi and dsRNAi had no more effect at 7 dpi. No phenotype (mortality, fecundity, rate of development) was observed in *dsA-crt-1 *injected aphids.

### Silencing of cathepsin-L

**Figure 3:**
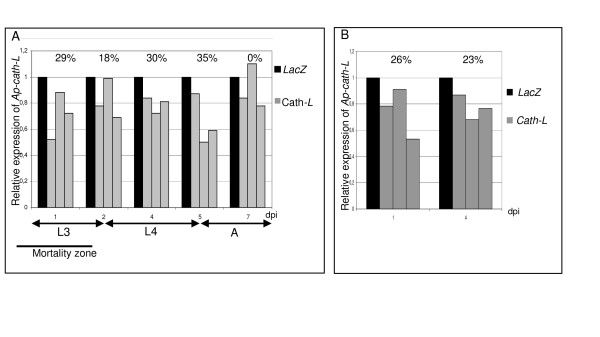
**Knockdown of *Ap-cath-L *expression**. *Ap-cath-L *mRNA level was measured in aphids injected with LacZ control dsRNA and with two distinct ds*Ap-cath-L*: ds1*Ap-cath-L *(A) and ds2*Ap-cath-L *(B). *Ap-cath-L *transcripts level is expressed as the proportion of level in Lac-Z injected control. Average percentages of *Ap-cath-L *gene expression are indicated for each dpi.

A similar experiment was performed to inhibit *Ap-cath-L *expression by injecting 353 nt dsRNAs (ds1*Ap-cath-L*) corresponding to the middle part of the CDS (Fig. 1) into L3 abdomens. As previously observed for calreticulin, approximately half of control and silenced aphids died, and a high variability was observed between replicates. Injection of ds1*Ap-cath-L *reduced *Ap-cath-L *expression by 29 ± 18% at 1 dpi, induced a maximum inhibition of 35 ± 19% at 5 dpi and was no more effective at 7 dpi (Fig. 3A). No clear phenotype was noticed. These results are similar to the *Ap-crt-1 *knockdown. A second *Ap-cath-L *dsRNA (ds2*Ap-cath-L*) (Fig. 1) including 124 nucleotides of the 5'end transcribed untranslated region plus the 316 nucleotides of the 5'end of the CDS, was injected in order to test the effect of the localisation of the dsRNA within the mRNA on RNAi efficiency (Fig. 3B). No significant difference of RNAi efficiency was obtained between ds1*Ap-cath-L *and ds2*Ap-cath-L*.

## Discussion

In this study, microinjection of long dsRNA was shown to transiently silence the expression of two marker genes in the pea aphid *A. pisum*. This work confirms the effectiveness of RNAi in various tissues of the pea aphid and demonstrates that long dsRNA can be substituted to siRNA to induce the inhibition of gene expression in aphids [18]. Since the two marker genes were transcribed all over the aphid life cycle, any development stage could be triggered to perform RNAi. Third stage instar (L3) was selected to initiate RNAi since it was the smallest and youngest stage adapted to injection in our hands. Our experiments were characterized by a high mortality within the two days following injection not related to gene silencing. This unspecific mortality was shown to be partly explained by the disruptive effect of the injection but also by the high injection volume used to deliver dsRNA (46 nl) in L3. No comparable mortality was observed following the injection of siRNAs but the injected volume was lower (5 nl) and the target stage was older (adult female) [18].

RNAi efficiency might depend on the targeted gene and its expression profile. We analysed the RNAi efficiency of two marker genes with different tissue expression patterns. For *Ap-crt *and *Ap-cath-L *genes, similar inhibition of gene expression from 30 to 40% was obtained. By injecting siRNAs in aphid females, Mutti et al. [18] obtained a decrease of 50% of the expression of a salivary gland gene. This suggests that the two different techniques (injection of long dsRNA *vs *siRNA) induce the same range of decrease of target gene expression. Moreover, these results demonstrate that different aphid tissues seem to be equally sensitive to gene knockdown. RNAi efficiency appears to be highly variable according to the targeted gene and insect species. The maximum depletion of approximately 40–50% of mRNAs obtained in aphid is not a high efficiency for RNAi compared to what was previously obtained in *Spodoptera litura *(95% of inhibition for a gene encoding an aminopeptidase) [15] or in *Drosophila melanogaster *(62% of inhibition for the *Drs *gene) [22]. However, similar efficiency has been recorded in the hemipterean insect *Rhodnius prolixus *[23] or in grasshopper [9]. For most of these cited works (including Mutti et al. [18] for the pea aphid), RNAi efficiency was tested on individuals rather than on pooled individuals as we did. Pooling individuals might have homogenised individual differences and the inhibition of 40% we observed, might reflect a mix of higher and lower RNAi efficiency. From our point of view, pooling individuals better reflects the general efficiency of RNAi. However, despite pooling of individuals and synchronization of the age of the individuals to be injected, quantification by qRT-PCR of RNAi efficiency revealed high variations between the replicates. Similar variations due to the detection method and to the variability of RNAi efficiency have been previously described for the apple moth *Epiphyas postvittana *[6].

Various parameters can be modified to increase RNAi efficiency. The most obvious parameter is to increase the amount of dsRNA delivered in the aphid. Injection of volumes higher than 46 nl has been tested but was not effective to increase RNAi efficiency since they generated very high mortality rates. In the hemipteran insect *Rhodnius prolixus*, the low RNAi efficiency has been increased by multiplying injections. Two successive injections of dsRNA resulted in an increase of RNAi efficiency from 38 to 75% of inhibition [23]. Similar double injections were performed in aphids but resulted in very high mortality preventing the estimation of RNAi efficiency (data not shown). RNAi efficiency can depend also on the sequence used as dsRNA against the targeted gene. Two different dsRNA were tested against the gut protease *Ap-cath-L*, each corresponding to either the central or the 3'part of the CDS. No difference in gene knockdown efficiency was observed, suggesting that in our experimental conditions, selecting different parts of the CDS of the target gene does not improve RNAi efficiency.

The duration of RNAi inhibition for cathepsin-L and calreticulin was similar, starting the first day following injection, reaching its maximum of 35–40% after five days and disappearing at seven days. During this period, aphids moult two times: starting in the third instar (L3) one day after injection, the knockdown continued at the fourth instar (L4) and ended after the aphids have reached the adult stage seven days later, suggesting that moulting does not disrupt RNAi effect. Gene knockdown of a salivary gland gene in the pea aphid had a different profile since inhibition was observed only 3 days after injection, but was maximal after 5 days, as in our conditions [18]. A similar duration of RNAi has been described for the *EposPBP1 *gene in apple moth [6] but duration of gene knockdown by RNAi appears to highly depend on insect species: RNAi can last up to 25 days in the honeybee *Apis mellifera *[8] or more than 4 months for the flour beetle *Tribolium castaneum *[24].

No clear phenotype (e.g. growth, life span, fecundity) was observed neither in *Ap-crt-1 *nor in *Ap-cath-L *silenced aphids. The absence of a striking phenotype in ds*Ap-crt-1 *aphid is not surprising since the only phenotype observed after inactivation of the calreticulin gene in *Drosophila melanogaster *was a hypersensitivity to diethylether and resistance to isoflurane, two anaesthetic compounds [25]. These conditions were not tested for the ds*Ap-crt1 *injected aphids. By contrast, the absence of phenotype in *Ap-cath-L *silenced aphids was more unexpected. The *A. pisum *cathepsin-L is highly identical to the cathepsin-L gene previously characterized in the cotton aphid *Aphis gossypii *and in the peach-potato aphid *Myzus persicae *[19,26]. In *M. persicae*, the inhibition of cathepsin activities by oryzacystatin (a cystein protease inhibitor) led to a moderate inhibition of aphid growth and fecundity [26]. The knockdown of *Ap-cath-L *was expected to generate a similar phenotype. The absence of phenotype in *Ap-cath-L *silenced aphids could be due to the relative efficiency of the silencing (30% of inhibition). However, oryzacystatin is a general cysteine protease inhibitor and is probably not specific of cathepsin-L [27]. The growth defect observed in *M. persicae *and *A. gossypii *might be due to the inhibition of other cathepsins.

## Conclusion

This work confirms the effectiveness of RNAi in the pea aphid and demonstrates that long dsRNA can be used instead of siRNA [18] to induce the inhibition of gene expression in aphids. By triggering a different developmental stage (L3 rather than adult) and genes expressed in different tissues, our study is complementary to the work published by Mutti et al. [18]. By using L3, this work extended aphid RNAi to include larval stage and showed that RNAi knockdown is active during the moulting process. Moreover, we demonstrated that salivary glands were not the only aphid tissue sensitive to RNAi since this technique was as effective for a ubiquitously expressed gene like *Ap-crt1 *as for a gene specifically expressed in the gut like *Ap-cath-L*. Gene knockdown by dsRNA or siRNA injection will be undoubtedly a valuable tool for the aphid community to investigate aphid gene functions and start reverse genetics.

## Methods

### Acyrthosiphon pisum

The holocyclic clone YR2 [28] of *Acyrthosiphon pisum *(the pea aphid) was reared and maintained as clonal individuals (parthenogenesis) on *Vicia fabae *at 18°C under a 16 h photoperiod. In order to synchronise injected L3, L1 produced by parthenogenetic adult females were collected every 24 h and placed on new plants. L3 were obtained from these L1 96 h after collection of L1.

### Cloning of calreticulin and cathepsin-L coding sequences

Total RNAs from parthenogenetic adults were extracted with the "SV Total RNA Isolation kit" (Promega). First strand cDNAs were produced from 5 μg total RNA using an AMV reverse transcriptase (Promega) following the supplier's instruction. DNA contaminations were removed by treating RNA extractions products with RNAse-free DNAse (Ambion). Partial coding sequences of *Ap-crt *and *Ap-cath-L *were obtained using the CalF1 (5'GGCCGACAAGGGTATACAGA3')-CalR2 (5'TTCGGCAGAACCCATATGTCT3') and CathF1 (5'TCAGTACATCGCCTTCTCTTCA)-CathR1 (5'CTGGATGCAACACCACAGTT3') primer pairs. Amplified fragment were purified (Gel Extraction kit QIAGEN), cloned into the pGEMTeasy vector (Promega) and two clones were sequenced in both strands for each sequence (Macrogen, South Corea). Nucleotidic sequences alignments were performed with the ClustalW software [29].

### Analysis of calreticulin and cathepsin-L transcription pattern

Total RNA was extracted from the four instar stages (L1, L2, L3 and L4) and from adult parthenogenetic and sexual females as described above. Steady state level of *Ap-crt-1 *and *Ap-cath-L *mRNA was analysed by RT-PCR by using the CalF1-CalR3 (5'AGTCCCAATCGGCTTCTA3') or CathF2 (5'TTTGGCTGGTGGTGATAGA3')-CathR2 (5'CCTCGGCTTCATATGGGTAA3') specific primer pairs, respectively.

### Screening genomic traces

As the complete genome sequencing for *A. pisum *is ongoing, the near-final set of traces were available but not assembled. We downloaded the data from the National Human Genome Research Institute – Baylor College of Medicine [30] and scanned the traces on a local server where the complete set of fasta sequences was database formatted. We used *A. pisum *cathepsin L or calreticulin deduced protein sequence as a query to search (with tblastn) identical or similar genes in the pea aphid genome. Examination of the blast reports indicated whether matching traces corresponded to a unique or to different gene sequences. Then, we used a home-made program to iteratively find and assemble matching traces (with CAP3 [31]), in order to reconstruct the gene model(s) for each gene copy and exactly determine numbers of paralogs and gene sequences.

### dsRNA synthesis and delivery

*Ap-crt-1 *and *Ap-cath-L *target sequences were amplified by RT-PCR by using the CalF1-CalR3 and CathF2-CathR2 primers, respectively. PCR products were purified by gel extraction (Qiagen) and 5 ng of purified products were used in a second PCR reaction by using the CalF1-CalR3, CathF2-CathR2 and CathF1 (TCAGTACATCGCCTTCTCTTCA) CathR3 (TATCACCACCAGCCAAACT) PCR primers pairs, each primer including the T7 sequence (TAATACGACTCACTATAGGG) at their 5' end. Amplified products were purified by gel extraction and 1 μg was used as template for *in vitro *transcription for dsRNA synthesis by using the Megascript T7 kit (Ambion). Both RNA strands were generated in the same amplification experiment. Transcription products were treated as indicated by the supplier and resuspended in RNAse-free water (Promega). Lac-Z dsRNA was produced by using the LacZT7F-R primer pairs on a LacZ fragment cloned into pGEMTeasy vector.

Aphids were immobilised on ice and 46 nl of purified dsRNA resuspended in water at 6 μg/μl were dorsally injected at slow speed in the middle of L3 abdomen using 3.5 Drummond needles and the Nanoinject II nanoinjector (Drummond scientific). For each gene (including LacZ), 80 to 100 L3 were injected. Injected aphids were placed on plants to recover and rear at 18°C with 16 h light per day for 1 to 7 days. Mortality was recorded and aphids frozen in liquid nitrogen and kept at -80°C before use.

### Real Time PCR

From 5 to 10 individuals aphids were collected at each time point from 1 to 7 days after injection. Total RNAs were extracted from whole aphids by using "SV Total RNA Isolation kit" (Promega). DNA contaminations were removed by treating RNA extractions products with RNAse-free DNAse (Ambion) and purified by phenol-chloroform. First strand cDNA synthesis was performed using 1 μg total RNA and AMV reverse transcriptase (Promega) according to the supplier's instructions. Real time RT-PCR reactions were performed on an ABI Prism 7700 (Applied Biosystem) using 1:20 diluted cDNAs and SYBR^® ^Green PCR Master Mix according to the manufacturer's instructions. For each gene, triplicate assays were performed from at least three independent biological experiments (consisting each of 5 to 10 individual aphids). Gene-specific primer pairs CalF4 (5'GCCAGAACACATTCCTGACC3')-CalR4 (5'ATCATTGCTGGTTCCCATTC3') and CathF6 (5'TGTTGGTTTCGGTTCTGACA3')-CalR6 (5'ATACCCTTCGTCTCCCCAAG3') were used to amplify *Ap-crt-1 *and *Ap-cath-L *respectively. Transcript levels were normalized to the *A. pisum *ribosomal RpL7 transcripts as an internal control [21]. Averages and standard deviations from qRT-PCR values were calculated between replicates (pools of 7 to 10 aphids).

## Abbreviations

EST, Expressed Sequence Tags; L3, Third instar; dsRNAi, double-stranded RNA interference; dsRNA, double-stranded RNA; siRNAs, small interfering RNAs; nt; Nucleotides.

## Competing interests

The author(s) declares that there are no competing interests.

## Authors' contributions

SJ designed, coordinated the study and wrote the manuscript. She carried out molecular biology experiments, and aphid injections. GLT participated in the qPCR and Southern-blot experiments. JB carried out rearing pea aphids. CR realised the *in silico *analysis of the pea aphid genomic traces. DT and GKC participated in the coordination of the study and in writing the manuscript. All authors read and approved the final manuscript.
